# Development and Application of a Multiplex Real-Time TaqMan qPCR Assay for the Simultaneous Detection of African Swine Fever Virus, Classical Swine Fever Virus, Porcine Reproductive and Respiratory Syndrome Virus, Pseudorabies Virus, and Porcine Circovirus Type 2

**DOI:** 10.3390/microorganisms13071573

**Published:** 2025-07-03

**Authors:** Dongdong Yin, Shuangshuang Xu, Yayun Liu, Hao Guo, Mengdie Lan, Lei Yin, Jieru Wang, Yin Dai, Xuehuai Shen, Kai Zhan, Xiaocheng Pan

**Affiliations:** 1 Anhui Provincial Key Laboratory of Livestock and Poultry Product Safety, Institute of Animal Husbandry and Veterinary Science, Anhui Academy of Agricultural Sciences, Livestock and Poultry Epidemic Diseases Research Center of Anhui Province, Hefei 230031, China; yindd160@163.com (D.Y.); liuyy20210120@163.com (Y.L.); yinlei1989@yeah.net (L.Y.); wangjr0317@126.com (J.W.); dalin2080@aaas.org.cn (Y.D.); xuehuaishen1986@126.com (X.S.); 2 College of Veterinary Medicine, Anhui Agricultural University, Hefei 230036, China; shuangshuangxu@stu.ahau.edu.cn; 3 Feixi Xian Agriculture and Rural Affairs Bureau, Feixi 231200, China; howrykuo@gmail.com; 4 Ningguo City Animal Health Supervision Institute, Ningguo 242300, China; 18395513902@163.com

**Keywords:** African swine fever virus, classical swine fever virus, pseudorabies virus, multiplex qPCR, differential diagnosis, TaqMan assay

## Abstract

Since its emergence in China in 2018, African swine fever virus (ASFV) has posed a severe threat to the pig farming industry due to its high transmissibility and mortality rate. The clinical signs of ASFV infection often overlap with those caused by other swine viruses such as classical swine fever virus (CSFV), porcine reproductive and respiratory syndrome virus (PRRSV), pseudorabies virus (PRV), and porcine circovirus type 2 (PCV2), making timely and precise diagnosis a considerable challenge. To address this, we established a TaqMan-based multiplex real-time quantitative PCR (qPCR) assay capable of simultaneously detecting ASFV, CSFV, PRRSV, PRV, and PCV2. Specific primer-probe sets were developed targeting conserved genomic regions: the ASFV *P72* gene, CSFV *5’UTR* region, PRRSV *ORF6*, PCV2 *cap* gene, and PRV *gB* gene. After thorough optimization, the assay demonstrated robust analytical performance, exhibiting strong target specificity with no cross-detection of non-target pathogens. The detection threshold was determined to be 10 copies/μL per virus, indicating high assay sensitivity. Repeatability analysis revealed low variability, with intra- and inter-assay coefficient of variation values remaining below 2.3%. When applied to 95 clinical samples, the multiplex assay yielded results that were fully consistent with those obtained using commercially available singleplex qPCR kits. In conclusion, the multiplex TaqMan qPCR method developed in this study is characterized by high specificity, sensitivity, and reproducibility. It provides a reliable and efficient diagnostic tool for the simultaneous detection and differential diagnosis of ASFV and other clinically similar viral infections in swine, thereby offering robust technical support for swine disease surveillance and control.

## 1. Introduction

African swine fever (ASF), induced by the African swine fever virus (ASFV)—a large, double-stranded DNA virus belonging to the *Asfarviridae* family—is a highly lethal disease of domestic and wild boars [1,2]. Following its initial outbreak in China in 2018, ASF has triggered widespread disruptions across the swine sector, causing profound economic consequences. The outbreaks have not only affected the health and productivity of swine herds but have also posed significant challenges to the livelihoods of pig farmers and the stability of national food security [3,4]. In addition to ASFV, other major viral pathogens threatening swine production include classical swine fever virus (CSFV), porcine reproductive and respiratory syndrome virus (PRRSV), pseudorabies virus (PRV), and porcine circovirus type 2 (PCV2) [5]. These pathogens often induce clinical signs and pathological changes that overlap with those of ASF, making differential diagnosis particularly challenging [6,7,8]. This issue is exacerbated by the increasing incidence of co-infections in intensive swine farming systems, complicating accurate etiological identification in clinical and post-mortem investigations.

Currently, diagnostic approaches for ASFV, CSFV, PRRSV, PRV, and PCV2 mainly rely on pathogen isolation, conventional polymerase chain reaction (PCR), and quantitative PCR (qPCR) [9,10,11,12,13,14]. Although qPCR is considered the “gold standard” for confirming infection, it is time-consuming, labor-intensive, and lacks the sensitivity needed for rapid diagnosis [15]. Moreover, most existing diagnostic protocols are designed to detect single pathogens, limiting their utility in differentiating between ASF and other clinically similar viral diseases. Multiplex real-time PCR (RT-PCR) based on TaqMan probe chemistry offers a powerful alternative for the simultaneous detection of multiple pathogens in a single reaction tube. This approach utilizes specific primers and fluorescent probes to enable parallel amplification and detection of distinct target genes, offering high specificity, sensitivity, and operational simplicity. As such, it represents an effective tool for improving diagnostic efficiency in swine disease surveillance.

This study aimed to establish and evaluate a TaqMan-based multiplex RT-PCR assay capable of concurrently identifying five major swine pathogens: ASFV, CSFV, PRRSV, PRV, and PCV2. Compared with conventional PCR, this assay eliminates the need for post-amplification electrophoresis, reducing labor and contamination risk, and is better suited for rapid, large-scale screening. This method is expected to provide accurate and reliable molecular diagnostic support for the detection of ASF and its clinically similar viral infections, thereby contributing to timely disease control and prevention strategies.

## 2. Materials and Methods

### 2.1. Primer and Probe Design, and Standard Plasmid Construction

The conserved regions of the *P72* gene of ASFV, the 5′ untranslated region (5′UTR) of CSFV, the *ORF6* gene of PRRSV, the *cap* gene of PCV2, and the *gB* gene of PRV were selected with reference to sequences obtained from the National Center for Biotechnology Information (NCBI) database [13,16,17]. Specific primers and TaqMan probes were designed accordingly (Table 1). Synthetic plasmid standards containing the corresponding target sequences for ASFV, CSFV, PRRSV, PRV, and PCV2 were constructed. All primers, probes, and plasmid standards were custom synthesized by General Biotechnology Co., Ltd. (Chuzhou, Anhui, China). The synthesized partial fragments of the ASFV *P72* gene, CSFV 5′UTR, PRRSV *ORF6*, PCV2 *cap* gene, and PRV *gB* gene (Table S1) were cloned into the pMD-19T plasmid (Takara, Beijing, China) to construct plasmid standards.

### 2.2. Optimization of Multiplex RT-PCR Conditions

A multiplex TaqMan-based RT-PCR assay was developed utilizing the five recombinant plasmids as templates. The multiplex real-time PCR reaction system is 20 μL: 2×PerfectStart^®^ II Probe qPCR SuperMix (BeijingTransGen Biotech Co., Ltd., Beijing, China) of 10 μL. Reaction conditions were optimized by testing eight concentrations of primers (0.12, 0.16, 0.20, 0.24, 0.28, 0.32, 0.36, and 0.40 pmol/μL) and eight concentrations of probes (0.08, 0.12, 0.16, 0.20, 0.24, 0.28, 0.32, and 0.36 pmol/μL) to determine the optimal concentration for each component, and RNase free H_2_O was supplemented to 20 μL. Temperature optimization was performed using a gradient PCR approach with annealing temperatures ranging from 46 °C to 60 °C. Five distinct fluorophores were employed for multiplex detection: FAM (ASFV), NED (CSFV), VIC (PRRSV), ROX (PCV2), and CY5 (PRV) (Table 1). The fluorescence signals were detected using an ABI 7500 Real-Time PCR System (Thermo Fisher Scientific, Waltham, MA, USA). The thermal cycling profile for the gradient experiment was as follows: initial denaturation at 95 °C for 30 s, followed by 45 cycles of 95 °C for 5 s and annealing/extension at the gradient temperatures (46–60 °C) for 34 s, with fluorescence signal acquisition during each extension step.

### 2.3. Construction of Standard Curves

Standard curves were established by applying a series of 10-fold dilutions of plasmid constructs corresponding to ASFV, CSFV, PRRSV, PRV, and PCV2, ranging from 1 × 10^8^ to 1 × 10^4^ copies/μL. Each dilution was amplified under the optimized qPCR conditions. The cycle threshold (Ct) values were plotted on the y-axis against the logarithm (base 10) of the plasmid copy number on the x-axis to construct standard curves for each target. The slope and correlation coefficient (R^2^) were calculated to examine the amplification efficiency and linearity of the assay.

### 2.4. Specificity, Sensitivity, and Reproducibility Evaluation

To assess the specificity of the multiplex RT-PCR assay, plasmid standards containing the target genes of ASFV, CSFV, PRRSV, PRV, and PCV2 were tested. In addition, nucleic acid templates from other common swine viruses, including porcine epidemic diarrhea virus (PEDV), transmissible gastroenteritis virus (TGEV), and porcine parvovirus (PPV), were included. Nuclease-free ddH_2_O served as the negative control (NC).

To examine the analytical sensitivity of the assay, a series of 10-fold serial dilutions (1 × 10^0^ to 1 × 10^8^ copies/μL) of mixed plasmid standards for ASFV, CSFV, PRRSV, PRV, and PCV2 were prepared. Each dilution was subjected to the multiplex qPCR under optimized conditions. The lowest concentration at which each target was consistently detected was defined as the limit of detection (LOD).

Reproducibility was assessed by analyzing three independent runs, each testing triplicates of plasmid standards at concentrations of 1 × 10^6^, 1 × 10^5^, and 1 × 10^4^ copies/μL. The coefficient of variation (CV, defined as the ratio of the standard deviation to the mean) was used to quantify variability. For intra-assay consistency, CV was calculated from triplicate measurements within a single run; for inter-assay consistency, CV was derived from results across three independent runs. CV values were computed as (standard deviation/mean) × 100%, ensuring objectivity in evaluating assay reproducibility.

### 2.5. Clinical Sample Testing

A total of 95 clinical samples were collected from pig farms in Anhui Province, China, comprising 44 blood samples, 6 brain samples, 15 liver samples, 10 lung samples, 10 spleen samples, and 10 kidney samples. For tissue samples, an appropriate amount was homogenized in PBS buffer using grinding tubes. Total nucleic acids were extracted from 200 μL of either tissue homogenate or serum using a viral DNA/RNA co-extraction kit (Beijing Joinstar Biomedical Technology Co., Ltd., Beijing, China). Reverse transcription was subsequently implemented utilizing the NovoScript^®^ Plus All-in-one 1st Strand cDNA Synthesis SuperMix (Suzhou Novoprotein Scientific Co., Ltd., Suzhou, China) to generate complementary DNA (cDNA). A total of 95 clinical samples were analyzed utilizing the multiplex RT-qPCR assay developed in this study. The reaction mixture for clinical sample screening (20 μL) comprised 10 μL 2×PerfectStart^®^ II Probe qPCR SuperMix, 0.32 pmol/μL of each primer (1 μL per 10 μM primer), 0.12 pmol/μL of each probe (1 μL per 10 μM probe), 2 μL template nucleic acid, and 5 μL nuclease-free water. For comparative evaluation, each specimen was also independently tested with five separate commercial TaqMan-based singleplex qPCR kits (Qingdao Lijian Biotechnology Co., Ltd., Qingdao, China), each specific for ASFV, CSFV, PRRSV, PRV, or PCV2. The diagnostic consistency between the multiplex method and the individual assays was assessed by comparing detection outcomes across all the platforms.

## 3. Results

### 3.1. Optimization of Multiplex RT-PCR Reaction Conditions

**Figure 1:**
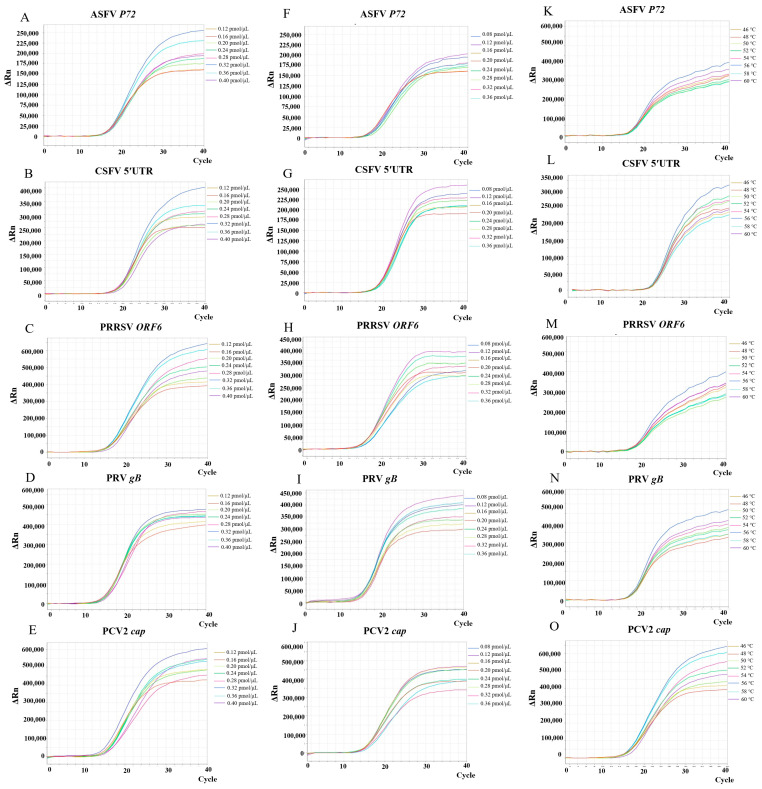
Optimization of fluorescence intensity for the multiplex qPCR assay. Effects of primer concentrations (0.12–0.40 pmol/μL) on fluorescence signals for ASFV (**A**), CSFV (**B**), PRRSV (**C**), PRV (**D**), and PCV2 (**E**) detection. Effects of probe concentrations (0.08–0.36 pmol/μL) on fluorescence signals for ASFV (**F**), CSFV (**G**), PRRSV (**H**), PRV (**I**), and PCV2 (**J**) detection. Effects of annealing temperature gradients (46–60°C) on fluorescence signals for ASFV (**K**), CSFV (**L**), PRRSV (**M**), PRV (**N**), and PCV2 (**O**) detection. Optimization was performed using recombinant plasmid standards as templates on an ABI 7500 Real-Time PCR System (Thermo Fisher Scientific, USA).

Different concentrations of primers and TaqMan probes were systematically assessed to refine the reaction parameters for the multiplex RT-PCR assay. As shown in Figure 1A–E, the optimal primer concentration for ASFV, CSFV, PRRSV, PRV, and PCV2 was determined to be 0.32 pmol/μL, while the optimal probe concentration for all five targets was 0.12 pmol/μL (Figure 1F–J). Temperature optimization was performed using a gradient ranging from 46 °C to 60 °C. The optimal annealing temperature for all five viruses was determined to be 56 °C, as this temperature yielded the highest fluorescence intensity (Figure 1K–O). These optimized parameters (0.32 pmol/μL primers, 0.12 pmol/μL probes, 56 °C annealing temperature) were used for all subsequent multiplex RT-PCR reactions.

### 3.2. Establishment of Standard Curves

**Figure 2:**
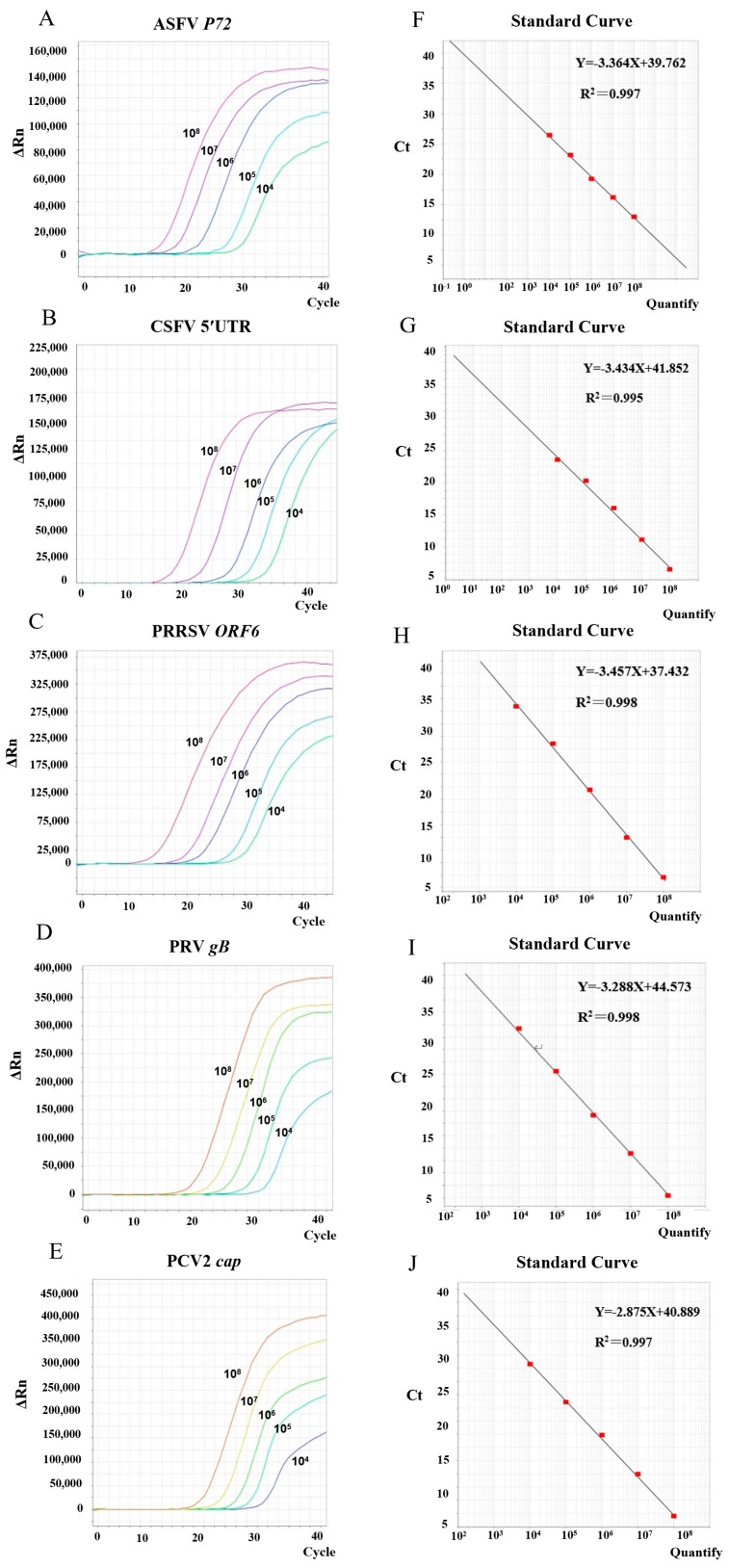
Detection standard curves of the multiplex real-time PCR method developed in this study. Under optimal amplification conditions, the optimized ASFV (**A**), CSFV (**B**), PRRSV (**C**), PRV (**D**), and PCV2 (**E**) amplification curves were generated using real-time PCR. Linear regression analysis of standard curve of ASFV (**F**), CSFV (**G**), PRRSV (**H**), PRV (**I**), and PCV2 (**J**) generated from 10-fold serial dilutions of plasmid standards (1 × 10^4^–1 × 10^8^ copies/μL).

Tenfold serial dilutions of plasmid standards for ASFV, CSFV, PRRSV, PRV, and PCV2 were prepared and utilized as templates under the optimized PCR conditions to construct standard curves. The linear regression analysis for each standard curve yielded the following results (Figure 2): ASFV: R^2^ = 0.997, slope = −3.364; equation: Y = −3.364X + 39.762; CSFV: R^2^ = 0.995, slope = −3.434; equation: Y = −3.434X + 41.852; PRRSV: R^2^ = 0.998, slope = −3.457; equation: Y = −3.457X + 37.432; PRV: R^2^ = 0.998, slope = −3.288; equation: Y = −3.288X + 44.573; PCV2: R^2^ = 0.997, slope = −2.875; equation: Y = −2.875X + 40.889. All R^2^ were greater than 0.99, indicating strong linear relationships between Ct values and the logarithm of template copy numbers. These data confirm the high quantification accuracy of the multiplex assay.

### 3.3. Specificity of the Multiplex RT-PCR Assay

**Figure 3:**
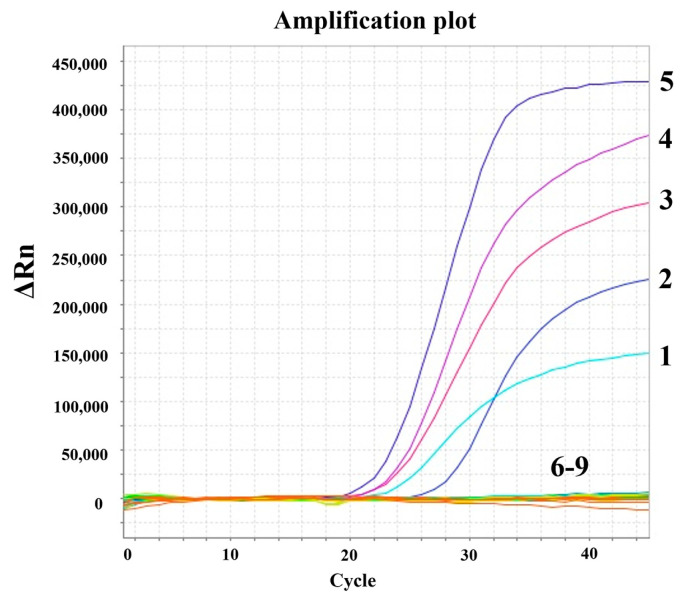
Specificity of the multiplex real-time PCR assay. Amplification curves were generated for the following targets: 1: ASFV; 2: CSFV; 3: PRRSV; 4: PCV2; 5: PRV; 6: Porcine epidemic diarrhea virus (PEDV); 7: TGEV; 8: PPV; 9: Nuclease-free H_2_O. No cross-detection was observed, as only target-specific plasmids generated amplification signals in their respective fluorescence channels (FAM/ASFV, NED/CSFV, VIC/PRRSV, ROX/PCV2, CY5/PRV), confirming primer-probe specificity. Tests were performed using plasmid standards for target viruses and extracted nucleic acids from non-target pathogens under optimized assay conditions (56 °C annealing temperature, 0.32 pmol/μL primers, 0.12 pmol/μL probes).

To evaluate the analytical specificity of the multiplex assay, nucleic acids from PEDV, TGEV, and PPV were tested alongside the five target pathogens. As depicted in Figure 3, only the ASFV, CSFV, PRRSV, PRV, and PCV2 templates produced positive amplification curves, while no amplification signals were noted for non-target viruses or the NC. These findings demonstrate the high specificity of the multiplex TaqMan-based qPCR assay, with no cross-reactivity observed.

### 3.4. Sensitivity of the Multiplex RT-PCR Assay

**Figure 4:**
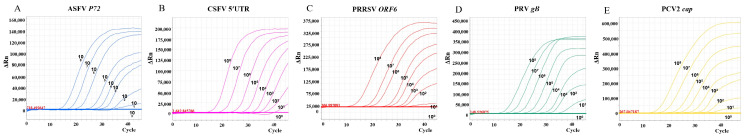
Sensitivity of the multiplex real-time PCR assay. Detection limit for (**A**) *P72* gene of ASFV; (**B**) 5′UTR of CSFV; (**C**) *OFR6* gene of PRRSV; (**D**) *gB* gene of PRV; (**E**) *cap* gene of PCV2. Serial 10-fold dilutions of mixed plasmid standards (1 × 10^0^–1 × 10^8^ copies/μL) were tested. The limit of detection was 10 copies/μL for all targets.

To assess the analytical sensitivity of the assay, serial 10-fold dilutions of the five plasmid standards were subjected to multiplex RT-PCR. As illustrated in Figure 4, the assay was capable of detecting all five target viruses at a minimum concentration of 1 × 10^1^ copies/μL.

### 3.5. Reproducibility of the Multiplex RT-PCR Assay

To evaluate the reproducibility of the developed multiplex RT-PCR assay, standard plasmids of ASFV, CSFV, PRRSV, PRV, and PCV2 were subjected to serial 10-fold dilutions and tested in three independent runs, each performed in triplicate. The results are presented in Table 2. The intra-assay CV for ASFV ranged from 0.341% to 1.854%, and the inter-assay CV ranged from 0.676% to 1.540%. For CSFV, the intra-assay CV ranged from 0.758% to 1.143%, and the inter-assay CV ranged from 0.143% to 1.097%. PRRSV showed intra-assay CVs between 0.911% and 2.076%, and inter-assay CVs between 0.487% and 1.304%. For PRV, the intra-assay CV ranged from 0.043% to 0.893% and the inter-assay CV from 0.175% to 1.638%. PCV2 exhibited intra-assay CVs ranging from 0.900% to 1.711% and inter-assay CVs from 1.286% to 2.232%. These results indicate that the developed assay demonstrates high stability and excellent reproducibility across different concentrations and test batches.

### 3.6. Detection of Clinical Samples

A total of 95 clinical samples—including blood and tissue samples—were collected from various swine farms across Anhui Province, China. The multiplex RT-PCR assay developed in this study was applied to assess all the clinical samples.

The assay detected no ASFV-positive samples (0/95). For other viruses, CSFV was detected in 3.16% (3/95) of samples, primarily in blood (2/3) and spleen (1/3); PRRSV in 8.42% (8/95), predominantly in lung (5/7) and blood (3/7); PCV2 in 12.63% (12/95), mainly in liver (6/12), blood (4/12), and kidney (2/12); and PRV in 2.11% (2/95), primarily in brain (2/2) (Table 3). Co-infections were identified in 8.42% (8/95) of samples: CSFV + PRRSV in 1.05% (1/95, blood sample) and PRRSV + PCV2 in 7.37% (7/95, primarily in lung (5/8) and liver (3/8)), reflecting the clinical relevance of mixed infections in swine herds. All the results were 100% concordant with commercial singleplex qPCR kits (Table 3), validating the assay’s accuracy. These findings suggest that the developed multiplex RT-PCR assay is suitable for preliminary clinical screening of ASF and other clinically similar viral infections in swine. Its accuracy and agreement with existing diagnostic platforms confirm its potential utility in field-based surveillance and laboratory diagnostics.

## 4. Discussion

ASFV, introduced into China in 2018, has severely impacted the pig farming sector, largely attributable to its aggressive transmission dynamics and high virulence [6,18,19]. In clinical settings, the early identification of ASFV is often complicated by its overlapping clinical manifestations with other economically important viral diseases, including CSF, PRRS, PR, and PCV2 infection. These similarities hinder rapid and accurate diagnosis, especially during early outbreak stages or in cases of mixed infections. In response to this diagnostic challenge, we developed a multiplex TaqMan-based RT-PCR assay capable of simultaneously detecting ASFV, CSFV, PRRSV, PRV, and PCV2. This method provides critical technical support for pathogen differentiation in complex clinical contexts and facilitates prompt, targeted disease control.

Conventional diagnostic methods for these viruses—including virus isolation, enzyme-linked immunosorbent assay, and immunofluorescence—are cost-effective but limited by long turnaround times and lower sensitivity [20,21,22,23,24]. RT-PCR techniques, particularly those utilizing TaqMan probes, have become the preferred diagnostic tools due to their high sensitivity, specificity, and rapid turnaround. However, most existing PCR-based assays target individual pathogens, limiting their utility in differential diagnosis involving co-infections. Several previous studies have reported sensitive singleplex or limited multiplex RT-PCR assays for swine viruses. For example, Li et al. developed a qPCR assay for ASFV with a LOD of 10^1^ copies/μL [25]. Tu et al. documented a TaqMan-based RT-PCR method for PRRSV, also achieving a LOD of 10^1^ copies/μL [26]. Ma established a quadruplex RT-qPCR assay for porcine respiratory coronavirus (PRCoV), PRRSV, swine influenza virus (SIV), and PRV, with LODs of 129.594, 133.205, 139.791, and 136.600 copies/μL, respectively [27]. Wang et al. developed a multiplex qPCR assay for PRRSV, PCV2, PCV3, and *Streptococcus suis*, achieving LODs of 2.80 × 10^1^, 1.96 × 10^2^, 2.30 × 10^2^, and 1.75 × 10^3^ copies/μL, respectively [17]. To date, no published study has reported a multiplex RT-PCR assay capable of simultaneously detecting ASFV, CSFV, PRRSV, PRV, and PCV2 in a single reaction. In this study, we successfully integrated five sets of highly specific primers and TaqMan probes into a single-tube reaction system. The use of distinct fluorophores (FAM, VIC, ROX, NED, and CY5) allowed for simultaneous amplification and signal discrimination of five different viral targets. This approach condenses what would otherwise require five separate singleplex assays into a single reaction, significantly enhancing diagnostic throughput and reducing time and cost. As a result, this multiplex assay is well suited for high-throughput screening and rapid diagnostics in routine surveillance and primary-level veterinary laboratories.

The multiplex TaqMan RT-PCR assay prepared in our study enables accurate and simultaneous detection of five major swine pathogens: ASFV, CSFV, PRRSV, PRV, and PCV2. In specificity evaluations, only nucleic acids from the target pathogens generated positive amplification signals, while those from non-target pathogens and NCs showed no amplification, confirming the high specificity of the assay and its utility for differential diagnosis. In terms of analytical sensitivity, the assay achieved a consistent LOD of 1 × 10^1^ copies/μL for all five target pathogens. This sensitivity exceeds that of conventional PCR methods and allows for the early detection of infections, thereby facilitating timely intervention and control measures. Moreover, the reproducibility of the assay was rigorously validated. Intra-assay and inter-assay CVs across multiple replicates remained below 2.3%, indicating high stability and reliability. These attributes make the assay highly suitable for routine diagnostic applications, offering robust and accurate performance in field and laboratory settings. The developed assay underwent clinical validation with 95 field samples, and its diagnostic concordance was assessed by parallel testing with established commercial singleplex RT-PCR assays. The results demonstrated full concordance between the multiplex and singleplex assays, further validating its diagnostic accuracy. Additionally, the multiplex assay successfully identified co-infections involving CSFV and PRRSV, as well as PRRSV and PCV2, highlighting its capacity to detect multiple pathogens simultaneously—a critical advantage in the context of mixed infections increasingly observed in swine production systems.

Despite the promising performance of the developed multiplex RT-PCR assay, several limitations should be acknowledged. Due to the absence of ASF outbreaks during sample collection periods, the absence of ASFV-positive clinical samples has constrained direct clinical validation of the assay for ASFV detection. Although the assay demonstrated high sensitivity and specificity using plasmid standards, further validation with genuine clinical ASFV samples from endemic regions is necessary to confirm its performance under real-world conditions. Future studies could expand the sample scope across multiple geographical regions to evaluate the assay’s efficacy against diverse viral strains. This approach would facilitate continuous optimization of the diagnostic protocol and enhance its application in porcine disease surveillance and clinical diagnostics.

## 5. Conclusions

In summary, this study presents a robust, sensitive, and specific multiplex TaqMan RT-PCR method for the simultaneous detection of ASFV, CSFV, PRRSV, PRV, and PCV2. The optimized reaction conditions are as follows: a 20-μL reaction volume containing 10 μL of 2×PerfectStart^®^ II Probe qPCR SuperMix, 0.32 pmol/μL of each primer set, and 0.12 pmol/μL of each TaqMan probe. The thermal cycling parameters include an initial denaturation at 95 °C for 30 s, followed by 45 cycles of 95 °C for 5 s and 56 °C for 34 s (for annealing/extension with fluorescence acquisition). The method offers a valuable diagnostic tool for the rapid and accurate differentiation of ASF and other clinically similar viral diseases in swine, offering strong technical support for early diagnosis, epidemiological surveillance, and targeted disease control strategies.

## Figures and Tables

**Table 1:** Primers and probes used in this study.

Name	Sequence (5′-3′) of Primer/Probe	Product Length (bp)
ASFV-F	ATCCGATCACATTACCTA	174
ASFV-R	GCTTCAAAGCAAAGGTAA	
ASFV-P	FAM-TTCCGTAACTGCTCATGGTATCAATCT-BHQ1	
CSFV-F	TAGCAAACGGAGGGAC	87
CSFV-R	CACGTCGAACTACTGAC	
CSFV-P	NED-CTCCCTGGGTGGTCTAAGTCCTGA-BHQ2	
PRRSV-F	CGGCAAATGATAACCAC	155
PRRSV-R	CCGTTGTTATTTGGCATA	
PRRSV-P	VIC-CGGCTCCACTACGGTCAACG-BHQ1	
PCV2-F	ATCGGAGGATTACTTCC	200
PCV2-R	CAGAGAATTTAATCTTAAAGACC	
PCV2-P	ROX-AAGAATGCTACAGAACAATCCACGG-BHQ2	
PRV-F	ACACCTACACCAAGATCG	107
PRV-R	GAAGGAGTCGTAGGGGTA	
PRV-P	CY5-CCTCCACCTCCTCGACGATG-BHQ2	

**Table 2:** Validation of the detection repeatability of the developed multiplex real-time PCR method.

Viruses	Standard Copies/uL	CT (Mean ± SD)		CT (Mean ± SD)	
		Intra-Assay	CV/%	Inter-Assay	CV/%
ASFV	1 × 10^4^	26.535 ± 0.278	1.047	26.389 ± 0.259	0.981
	1 × 10^5^	22.496 ± 0.417	1.854	22.627 ± 0.153	0.676
	1 × 10^6^	18.766 ± 0.064	0.341	18.504 ± 0.285	1.540
CSFV	1 × 10^4^	28.744 ± 0.218	0.758	28.715 ± 0.041	0.143
	1 × 10^5^	25.715 ± 0.294	1.143	25.517 ± 0.280	1.097
	1 × 10^6^	21.408 ± 0.188	0.878	21.475 ± 0.094	0.438
PRRSV	1 × 10^4^	25.564 ± 0.233	0.911	25.451 ± 0.124	0.487
	1 × 10^5^	21.466 ± 0.278	1.295	21.319 ± 0.207	0.971
	1 × 10^6^	17.535 ± 0.364	2.076	17.484 ± 0.228	1.304
PRV	1 × 10^4^	24.824 ± 0.133	0.536	24.639 ± 0.206	0.836
	1 × 10^5^	21.728 ± 0.194	0.893	21.757 ± 0.038	0.175
	1 × 10^6^	18.820 ± 0.008	0.043	18.503 ± 0.303	1.638
PCV2	1 × 10^4^	24.486 ± 0.275	1.123	24.335 ± 0.313	1.286
	1 × 10^5^	20.509 ± 0.351	1.711	20.396 ± 0.269	1.319
	1 × 10^6^	17.782 ± 0.160	0.900	17.649 ± 0.394	2.232

**Table 3:** Results of multiplex real-time PCR methods detecting the clinical samples.

Viruses	Multiplex Real-Time PCR Methods		Commercial Singleplex qPCR Kits		Matrix Distribution (*n*)
	Positive Samples	Positive Detection Rates (%)	Positive Samples	Positive Detection Rates (%)	
ASFV	0	0	0	0	
CSFV	3	3.16	3	3.16	Blood (2); Spleen (1)
PRRSV	8	8.42	8	8.42	Lung (5); Blood (3)
PRV	2	2.11	2	2.11	Brain (2)
PCV2	12	12.63	12	12.63	Liver (6); Blood (4); Kidney (2)
CSFV + PRRSV	1	1.05	1	1.05	Blood (1)
PRRSV + PCV2	7	7.37	7	7.37	Lung (4); Blood (3)

## Data Availability

The original contributions presented in this study are included in the article/Supplementary Materials. Further inquiries can be directed to the corresponding authors.

## Supplementary Materials

The following supporting information can be downloaded at: https://www.mdpi.com/article/10.3390/microorganisms13071573/s1, Table S1: Synthetic viral gene sequence information.

## Abbreviations

The following abbreviations are used in this manuscript:

ASF	African swine fever
ASFV	African swine fever virus
CSFV	Classical swine fever virus
PRRSV	Porcine reproductive and respiratory syndrome virus
PRV	Pseudorabies virus
PCV2	Porcine circovirus type 2
PCR	Polymerase chain reaction
Ct	Cycle threshold
R^2^	Correlation coefficient
