# Supplementary material for: Development and Application of a Multiplex Real-Time TaqMan qPCR Assay for the Simultaneous Detection of African Swine Fever Virus, Classical Swine Fever Virus, Porcine Reproductive and Respiratory Syndrome Virus, Pseudorabies Virus, and Porcine Circovirus Type 2
Source: Microorganisms. 2025 Jul 3;13(7):1573. doi: 10.3390/microorganisms13071573 (PMC12299348; doi:10.3390/microorganisms13071573)
Supplement: Supplementary file 1 [file microorganisms-13-01573-s001.zip › microorganisms-3679318-supplementary.pdf]

Table S1. Synthetic viral gene sequence information

Gene	Sequence
ASFV *p72*	cttccagacgcatgttcatctatatctgatattagccccgttacgtatccgatcacattacctattattaaaaacatttccgtaactgctc<br>atggtatcaatcttatcgacaaatttccatcaaagttctgcagctcttacatacccttccactacggaggcaatgcgattaaaacccc<br>cgatgatccgggtgcgatgatgattacctttgctttgaagccacgggaggaataccaacccag
CSFV 5′UTR	gctagccatgccctcagtaggactagcaaacggagggactagccgtagtggcgagctccctgggtggtctaagtcctgagtac<br>aggacagtcgtcagtagttcgacgtgagcagaagcccacctcgatatgctatgtggacgagggcatgcccaagacacaccttaa<br>ccctagcgggggtcgctagggtgaaatcgcatcacgtgatgggagtacgacctgatagggtgctgcagaggcccactattag
PRRSV *ORF6*	agatgccgtttgtgcttgctaggccgcaagtacattctggcccctgcccaccacgttgaaagtgccgcaggctttcatccgattgcg<br>gcaaatgataaccacgcatttgtcgtccggcgtcccggctccactacggtcaacggcacattggtgcccgggttgaaaagcctcg<br>tgttgggtggcagaaaagctgttaaacagggagtggtaaaccttgtcaaatatgccaaataacaacggcaagcagcagaaga<br>gaaagaagggggatggccagccagtcaatcagctgtgccagatgctgggtaagatcatcgctcagcaaaaccagtccagagg<br>c
PRV *gB*	Ccacatctactacaagaacgtcatcgtcacgaccgtgtggtccgggagcacgtacgcggccatcacgaaccgcttcacggacc<br>gcgtgcccgtccccgtgcaggagatcacggacgtgatcgaccgccgcggcaagtgcgtctccaaggccgagtacgtgcgcaa<br>caaccacaaggtgaccgccttcgaccgcgacgagaaccccgtcgaggtggacctgcgcccctcgcgcctgaacgcgctcggca<br>cccgcggctggcacaccaccaacgacacctacaccaagatcggcgccgcgggcttctaccacacgggcacctccgtcaactgca<br>tcgtcgaggaggtggaggcgcgctccgtgtacccctacgactccttc
PCV2 *cap*	cagaagcgtgattggaagaccaatgtacacgtcattgtggggccacctgggtgtggtaaaagcaaatgggctgctaattttgca<br>gacccggagaccacatactggaaaccacctagaaacaagtggtgggatggttaccatggtgaagaagtggttgttattgatgac<br>ttttatggctggctgccgtgggatgatctactgagactgtgtgaccgatatccattgactgtagagactaaaggtggaactgtacc<br>tttttttggcccgcagtattctgattaccagcaatcagaccccgttggaatggtactcctcaactgctgtcccagctgtagaagctctct<br>atcggaggattacttccttggtattttggaagaatgctacagaacaatccacggaggaagggggccagttcgtcaccctttccccc<br>ccatgccctgaatttccatatgaaataaattactgagtctttttttatcacttcgtaatggtttttatttttcatttagggtttaagtggggg<br>gtctttaagattaaattctctgaattgtacatacatggttacacggat
